# Lung cancer presenting with neurocognitive decline: a compelling case report highlighting healthcare seeking behavior in rural Nepal

**DOI:** 10.1097/MS9.0000000000001203

**Published:** 2023-08-17

**Authors:** Shailendra Katwal, Aastha Ghimire, Bharosha Bhattarai, Nishant Joshi

**Affiliations:** a Department of Radiology, Dadeldhura Subregional Hospital, Dadeldhura; b Patan Academy of Health Sciences, Lalitpur; c B.P Koirala Institute of Health Sciences, Dharan, Nepal

**Keywords:** case report, lung cancer, neurocognitive decline, healthcare seeking behavior

## Abstract

**Introduction:**

Lung cancer is a major contributor of burden of disease globally. Early diagnosis plays a crucial role in determining the patient’s prognosis. However, diagnostic constraints and healthcare-seeking behavior in rural areas contribute to the increased mortality and morbidity associated with this disease.

**Case presentation:**

The authors present a case that came in with mood and personality changes who was diagnosed with lung cancer with metastasis in the brain and did not seek health care services despite a prolonged duration of her neurocognitive symptoms.

**Clinical discussion:**

Rural communities face multiple barriers to early diagnosis and treatment, including healthcare-seeking behavior. It is crucial to develop policies aimed at addressing these barriers in order to enhance the health status of rural Nepal.

**Conclusion:**

The presentation of lung cancer with atypical symptoms can contribute to delays in diagnosis and treatment, ultimately impacting the overall prognosis of the patient. Late presentation to healthcare centers further exacerbates the burden of the disease, emphasizing the critical importance of proper healthcare-seeking behavior.

## Introduction

Highlights

- Lung cancer is a major contributor of global burden of disease whose prognosis worsens with delay in diagnosis.
- Patients in rural Nepal still prefer traditional and spiritual healers as their first point of contact for healthcare.
- Improving healthcare seeking behavior is as important as improving access to diagnostic and therapeutic modalities in these areas.

Cancer ranks as a leading cause of death and an important barrier to increasing life expectancy in every country of the world^1^. Lung cancer is the leading cause of cancer morbidity and mortality in men, whereas, in women, it ranks third for incidence, after breast and colorectal cancer, and second for mortality, after breast cancer^1^. Many of the symptoms of lung cancer are nonspecific and their onset is gradual. Therefore, early detection and timely curative treatment remains a challenge^2^. Diagnosis at early stages leads to more treatment options and better outcomes for the patients^3^. Rural Nepal has care-seeking behavior, rooted in a naturalistic lifestyle, and is community focused. So, a better understanding of the perceptions of the rural Nepali is crucial in advocating for sustainable and culturally-sensitive delivery of healthcare^4^. This case report has been reported in accordance with the CARE criteria^5^.

### Case details

A 51-year-old woman, who is a smoker, was accompanied by her family members to the outpatient department of our hospital. They reported that she had been experiencing right-sided weakness in her upper and lower limbs for the past 6 days. Additionally, she had decreased sensation on the right side of her body during the same duration. The patient denied any history of slurred speech, abnormal body movements, loss of consciousness, headaches, trauma, or vision. She had no known comorbidities. While exploring her symptoms further, the patient revealed that she had been experiencing a vague discomfort in her chest for approximately a year. Her family members further elaborated that she frequently complained of similar vague symptoms. Over the past few months, they have noticed changes in her mood and personality. Specifically, she had become forgetful, withdrawn, and exhibited rapid mood changes. She had instances when she could not remember the name of family members and forgotten to perform activities of daily routine. She was found wandering in a familiar neighborhood more than twice. Family members also mentioned a lack of coordination and clumsiness along with frequent mood swings and angry outbursts. The family members attributed all her symptoms to some spiritual disturbance and hence had been taking her regularly to Dhami Jhakris who are the ʻspiritual healersʼ of the locality. She was never taken to a healthcare center for the evaluation of these symptoms.

During the examination, the patient was conscious but appeared cachectic, exhibiting noticeable pallor. Decreased pain and temperature sensation were observed in the right upper and lower limbs. The patient’s muscle power was evaluated using the Medical Research Council (MRC) scale, yielding a score of 2 for the muscles of the right upper and lower extremities. Additionally, the patellar reflex on the right was diminished and graded as 1. She had decreased neurocognitive features, as evidenced by the Mini-Mental State Examination(MMSE) score of 18/30. Specific areas of concern included deficits in memory, attention, and processing speed.

**Figure 1:**
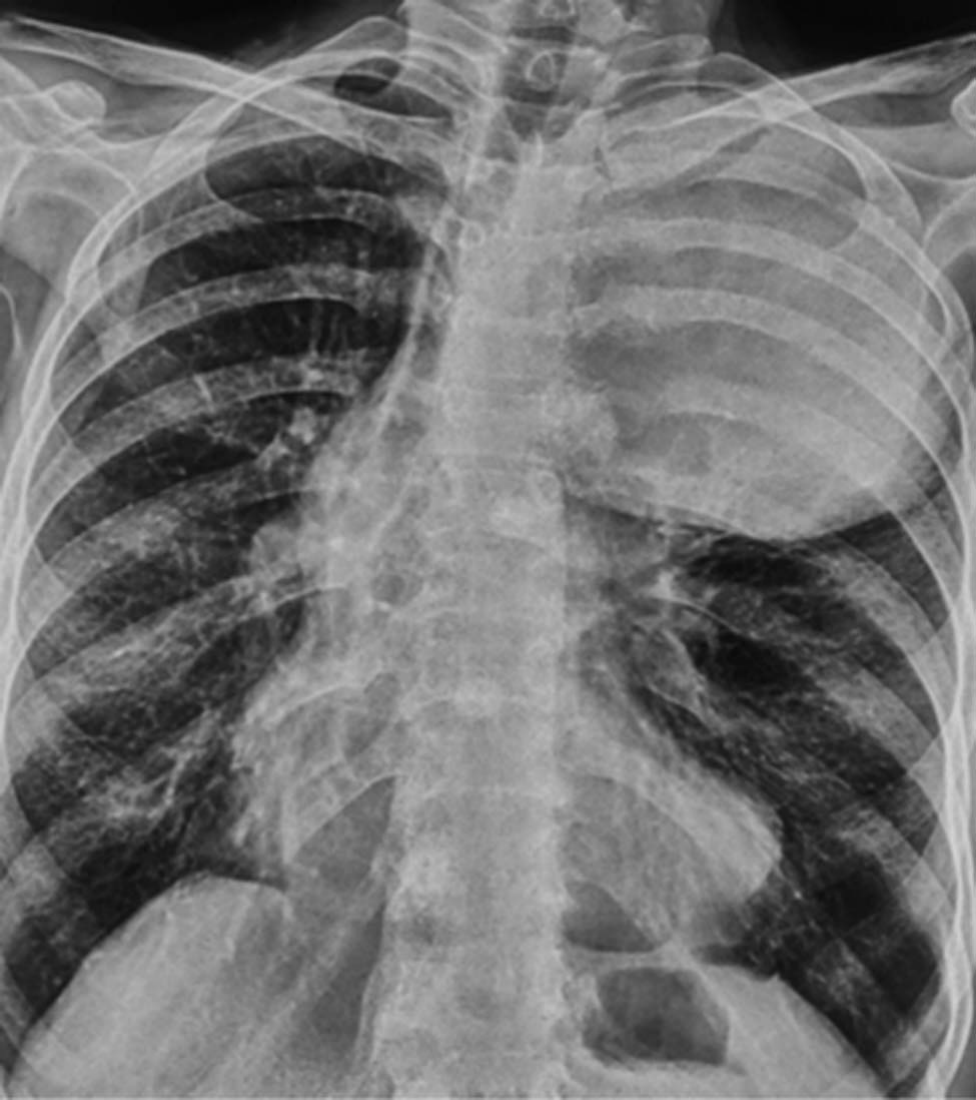
Chest radiograph posteroanterior view showing the relatively well-defined radio-opaque mass in the left upper and middle zone with mild displacement of trachea to right side.

**Figure 2:**
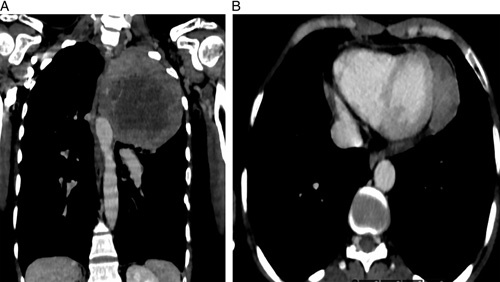
(A) Contrast-enhanced computed tomography coronal view showing thick-walled cavitary heterogeneously enhancing mass in left upper lobe. (B) Contrast-enhanced computed tomography axial view at the level of heart showing heterogenously enhancing soft tissue density mass in left lateral wall of pericardium.

**Figure 3:**
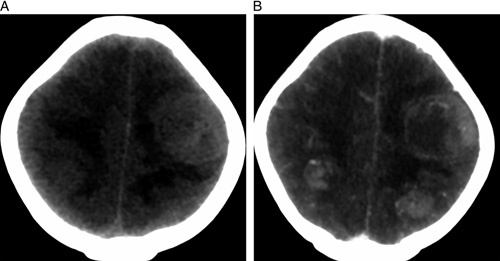
(A) Noncontrast computed tomography head axial view at the high frontal level showing isodense relatively ill-defined mass in bilateral frontal lobe (left>right) with surrounding edema. (B) Contrast-enhanced computed tomography head axial view at the high frontal level showing multiple heterogeneously enhancing masses in bilateral frontal lobe (left>right).

Based on the aforementioned findings, an emergency noncontrast computerized tomography (NCCT) of the head was performed. The results revealed an isodense and relatively ill-defined mass in the bilateral frontal lobe (Fig. 1A). To further evaluate this, a contrast-enhanced CT head was conducted, which demonstrated multiple heterogeneously enhancing masses in the bilateral frontal lobe (Fig. 1B). These unexpected findings were alarming and diverged from what would be anticipated in a vascular event. Subsequently, a comprehensive systemic examination was conducted, revealing a notable decreased air entry over the left upper chest. Following this, a complete blood count (CBC), renal function test (RFT), and a chest radiograph were performed. The chest radiograph revealed the presence of a radio-opaque mass in the left upper and middle zone (Fig. 2). Consequently, a contrast-enhanced computerized tomography (CECT) of the chest was requested for further evaluation. The CECT revealed a thick-walled cavitary mass in the left upper lobe, exhibiting heterogeneous enhancement (Fig. 3A). Additionally, it indicated the presence of a heterogeneously enhancing soft tissue density mass in the left lateral wall of the pericardium (Fig. 3B). Based on these findings, a working diagnosis of lung carcinoma with brain metastasis was established.

The patient, accompanied by family members, received the unfortunate news and was informed that the diagnosed condition was responsible for both the current and past symptoms. Initially, the patient and the family were skeptical about the diagnosis, as the patient had no respiratory symptoms and previous symptoms were vague. The family had considered the changes in her cognition as a matter of disturbances in spiritual wellbeing. However, after being given some time and additional counseling regarding the disease, diagnosis, prognosis, and treatment options, they eventually accepted the diagnosis. It was made clearer to the family that change in cognition can be the manifestation of many organic conditions and hence the decline in cognition in adults must be evaluated as early as possible. Together, the patient and her family made the decision to forego additional investigations and treatment. The family members were provided with information regarding the potential complications and progression of the disease, and were advised to focus on ensuring the patient’s comfort. The hospital offered assistance in the upcoming days to alleviate any discomfort and manage potential pain. The patient is currently attending follow up once a month with the support of her family members for assessing symptoms and for counseling. She has accepted her diagnosis. She is satisfied with her current quality of life with the help of her family.

## Discussion

Lung cancer is the second most commonly diagnosed cancer in females and has remained the leading cause of cancer related deaths worldwide^1^. These high rates of mortality reflect to late presentation mostly due to nonspecific disease presentation^2^. The common presenting symptoms of lung cancer include persistent cough, haemoptysis, dyspnea, chest pain, and weight loss^2^. However, symptoms at presentation may depend on other factors like the degree of invasion, metastasis, paraneoplastic syndromes, etc., and hence mimic with other conditions. So, lung cancer is still being diagnosed after the patient presents to the hospital due to some emergency condition and such patients are five times more likely to die within 1 year of presentation compared to those patients who are diagnosed during office visits^3^. In this particular case, the patient experienced a persistent sense of vague chest discomfort for an extended period. However, aside from this symptom, she did not exhibit any other typical indications. Notably, the patient also had a notable history of mood and personality changes, which should have raised concerns and prompted further evaluation. Unfortunately, individuals and families from rural, low-income areas often delay seeking mental health treatment until symptoms have reached to a more severe presentation. This is due to both a lack of proper knowledge as well as mental illness stigma^6^. The use of traditional and complementary medicine is associated with various factors such as education, residence, cancer stage, and perceived disease severity^8^. In rural Nepal, the preference for traditional and spiritual healers persists among the population when it comes to addressing their health concerns. Patients typically arrive at the hospital when their condition fails to improve despite repeated visits to these healers or when they experience acute distressing symptoms. In the case of this patient and her family, they attributed both her mood symptoms and prolonged vague symptoms to a perceived spiritual disturbance, leading them to seek care from a spiritual healer until the patient developed isolated motor symptoms. This highlights the prevailing health-seeking behavior in rural Nepal. The care-seeking behavior and the eventual choice between modern-based and belief-based medicine by a patient is affected by a number of considerations like the ease of its accessibility, the cost of services, their prior knowledge related to the illness, their belief system, and the severity of the medical situation^4^. In rural areas, individuals often face financial constraints that lead them to prioritize other aspects of life, such as work, before seeking assistance for health conditions that do not appear immediately alarming. In this case, the patient was brought to the hospital only after her symptoms began significantly impacting the daily life of the family. The development of motor symptoms necessitated help from another family member with day-to-day activities. Despite this, the patient and her family chose to wait for a week, hoping the symptoms would resolve on their own. Eventually, they presented to the hospital when the symptoms persisted.

Most people in rural Nepal still rely on traditional healers for their primary health care needs, not only because health facilities in rural areas are poorly functioning but also because these healers meet various healthcare needs^7^. The kind of traditional medicine provided by traditional healers (such as herbalists, bone setters, faith healers, and traditional midwives) is much more accessible to them than the practitioners of biomedicine and scholarly traditional medicine (such as Ayurveda, Unani, and Homeopathy)^8^. However, reliance on these modalities of treatment completely might lead to serious and potentially treatable diagnoses to be under looked. While public awareness is an integral part of addressing the gap in healthcare seeking behavior, one of the ways to make it efficient in such rural settings can be the inclusion of such traditional healers in the healthcare system and collaboration with their practices. This can be an important way to not to miss patients like ours who go through prolonged symptoms only to be diagnosed at the late stages of diseases.

The treatment of brain metastases has become increasingly individualized over the past decades and includes surgical approaches and radiation therapy^9^. Unfortunately, the treatment of such conditions in rural Nepal remains far from reality due to issues of accessibility and affordability. Many individuals endure chronic vague symptoms without ever receiving a proper diagnosis, primarily due to the lack of diagnostic facilities and specialist healthcare services. In the case of this patient, who presented with stroke-like features and long-standing mood symptoms in the emergency department, a diagnosis of lung cancer with brain metastasis was made. While the diagnosis did not offer a cure, it allowed the physician to initiate discussions about palliative and end-of-life care with both the patient and their family, which would not have been possible without proper diagnostic tools.

## Conclusion

Despite advancements in healthcare knowledge and services worldwide, individuals residing in rural Nepal continue to face significant barriers in accessing basic diagnostic and therapeutic interventions. Economic and social factors further contribute to the constraints in seeking appropriate care. For over half of the rural population, spiritual healers serve as the primary point of contact for healthcare needs. Recognizing these limitations in diagnosing and treating diseases with high morbidity and mortality rates is of paramount importance. Implementing awareness programs, ensuring access to diagnostic, and therapeutic interventions, and involving traditional healers in healthcare system policies can be crucial steps towards addressing these challenges.

## Ethical approval

Ethical approval is not required for case reports in my institution (Patan Academy of Health Sciences, Bagmati Lalitpur) so ethical approval was exempted.

## Consent

Written informed consent was obtained from the patient for the publication of this case report and accompanying images. A copy of written consent is available for review by the Editor-in-Chief of this journal on request.

## Author contribution

S.K.: conceptualization, mentor and reviewer for this case report and for data interpretation; A.G.: contributed in performing literature review, writing the paper and editing; B.B.: contributed in writing the paper; N.J.: contributed in writing the paper. All authors have read and approved the manuscript.

## Conflicts of interest disclosure

The authors declare that they have no conflicts of interest.

## Research registration unique identifying number (UIN)

1. Name of the registry: not applicable.
2. Unique identifying number or registration ID: not applicable.
3. Hyperlink to your specific registration (must be publicly accessible and will be checked): not applicable.

## Guarantor

Shailendra Katwal.

## Data availability statement

Not applicable.

## Provenence and peer review

Non commissioned, externally peer reviewed.
